# Charcot-Marie-Tooth 2b associated Rab7 mutations cause axon growth and guidance defects during vertebrate sensory neuron development

**DOI:** 10.1186/s13064-016-0058-x

**Published:** 2016-01-20

**Authors:** Olga Y. Ponomareva, Kevin W. Eliceiri, Mary C. Halloran

**Affiliations:** Department of Zoology, University of Wisconsin, 1117 West Johnson St., Madison, WI 53706 USA; Department of Neuroscience, University of Wisconsin, 1111 Highland Ave, Madison, WI 53705 USA; Neuroscience Training Program, University of Wisconsin, 1111 Highland Ave, Madison, WI 53705 USA; Medical Scientist Training Program, University of Wisconsin, 750 Highland Ave, Madison, WI 53705 USA; Laboratory for Optical and Computational Instrumentation, University of Wisconsin, 1675 Observatory Dr, Madison, WI 53706 USA

**Keywords:** CMT2b, Axon guidance, Axon branching, Axon transport, Endosome trafficking, Rab7, Zebrafish, Neurodegeneration

## Abstract

**Background:**

Charcot-Marie-Tooth2b (CMT2b) is an axonal form of a human neurodegenerative disease that preferentially affects sensory neurons. CMT2b is dominantly inherited and is characterized by unusually early onset, presenting in the second or third decade of life. Five missense mutations in the gene encoding Rab7 GTPase have been identified as causative in human CMT2b disease. Although several studies have modeled CMT2b disease in cultured neurons and in *Drosophila*, the mechanisms by which defective Rab7 leads to disease remain poorly understood.

**Results:**

We used zebrafish to investigate the effects of CMT2b-associated Rab7 mutations in a vertebrate model. We generated transgenic animals expressing the CMT2b-associated mutant forms of Rab7 in sensory neurons, and show that these Rab7 variants cause neurodevelopmental defects, including defects in sensory axon growth, branching and pathfinding at early developmental stages. We also find reduced axon growth and branching in neurons expressing a constitutively active form of Rab7, suggesting these defects may be caused by Rab7 gain-of-function. Further, we use high-speed, high-resolution imaging of endosome transport in vivo and find that CMT2b-associated Rab7 variants cause reduced vesicle speeds, suggesting altered transport may underlie axon development defects.

**Conclusions:**

Our data provide new insight into how disease-associated alterations in Rab7 protein disrupt cellular function in vertebrate sensory neurons. Moreover, our findings suggest that defects in axon development may be a previously unrecognized component of CMT2b disease.

## Background

Charcot-Marie-Tooth2b (CMT2b) is an axonal form of peripheral neuropathy characterized by loss of sensation in multiple somatosensory modalities, motor abnormalities, and a very early disease onset in the second or third decade of life [1, 2]. CMT2b is an autosomal dominant disease, caused by five missense mutations in the Rab7 gene (L129F, K157N, N161I, N161T, V162M) [3–6]. Rab7 is a small GTPase associated with late endosomal membrane compartments that has known roles in conversion of early endosomes to late endosomes, biogenesis of lysosomes, and maturation of autophagosomes [7, 8]. In addition, Rab7 has been shown to regulate retrograde trafficking, signaling and lysosomal degradation of neurotrophin receptors [7–11]. Like other GTPases, Rab7 cycles between a membrane-bound, active, GTP-bound form, and a cytosolic, inactive, GDP-bound form. CMT2b-associated amino acid substitutions occur in the proximity of the GTP-binding pocket and hydrolysis domains, and affect GDP and GTP exchange, increasing both Rab7 activation and hydrolysis-dependent inactivation, resulting in a form of Rab7 that is prone to remain in the active, GTP-bound form [12–14]. This finding, together with the dominant inheritance of the human disease, has led to the hypothesis that the disease is caused by overactivity of Rab7. However, several lines of evidence suggest the mutations do not cause simple gain of Rab7 function, but rather more complex alterations in function. Rab7 proteins containing the CMT2b-associated amino acid substitutions (hereafter referred to as CMT2b Rab7 mutants) show significantly lower affinity for both GDP and GTP [13], suggesting they could have reduced function. Indeed, when CMT2b Rab7 mutants are expressed in *Drosophila* photoreceptors, they are inefficiently recruited to endosomes, consistent with reduced function [15]. In contrast, although the CMT2b Rab7 mutants do cycle between membrane-associated and cytosolic states, they exhibit decreased ability to disassociate from the membrane, which leads to increased activity and augments interaction with several Rab7 effectors [14]. Moreover, the mutations do not interfere with the ability of Rab7 to bind its effector RILP and in some cases CMT2b Rab7 mutants are able to rescue Rab7 loss of function [12, 13, 15], which suggests retention of some wildtype function.

Several studies have investigated mechanisms by which the CMT2b mutations lead to disease by expressing CMT2b Rab7 mutants in cell lines or cultured neurons. CMT2b Rab7 mutants disrupted NGF signaling, NGF-induced neurite outgrowth, and trafficking of TrkA receptors in PC12 cells and DRG neurons [11, 16], suggesting that loss of neurotrophic support may contribute to CMT2B disease. In addition, expression of CMT2b Rab7 mutants in established DRG cultures caused neurite loss, indicating an axon degeneration effect [11]. Expression of CMT2b Rab7 mutants caused decreased neurite outgrowth in neuroblastoma cells, which could be reversed by treatment with valproic acid, an activator of extracellular signal-regulated kinase (ERK) and c-Jun terminal kinase (JNK) signaling pathways, both of the mitogen activated protein kinase (MAPK) superfamily [17, 18], suggesting this signaling pathway plays a role in the disease mechanism. Two recent studies expressed CMT2b Rab7 mutants in *Drosophila* to model the disease [15, 19]. One of these studies found that overexpression of CMT2b Rab7 mutants had no detectable effects on motor neuron or photoreceptor function, and suggested the CMT2b disease effects are due to partial loss of Rab7 function [15]. The second study showed that expression of the L129F CMT2b variant in *Drosophila* sensory neurons causes reduced pain and temperature sensation and is consistent with dominant effects of the mutant variants [19]. However, to date no vertebrate CMT2b models have been developed for in vivo analysis of the long sensory axons analogous to those affected in human disease.

Analyses of the transport dynamics of vesicles containing the CMT2b Rab7 mutants have also given varied results. CMT2b Rab7 mutants caused increased transport rates of vesicles in DRG neurons, an effect that was mimicked by a constitutively active Rab7, Q67L (CA-Rab7) [11]. Expression of CMT2b Rab7 mutants in *Drosophila* sensory neurons did not alter endosome speed but did result in fewer stationary vesicles [19]. Overall, the diverse and sometimes contradictory findings from studies in different systems and cell types indicate that the CMT2b Rab7 mutants have complex effects on the cell biology of neurons, and highlight the need for additional models for analysis of disease mechanism.

We developed a vertebrate model of CMT2b by expressing CMT2b Rab7 mutants in zebrafish spinal sensory neurons in vivo. The zebrafish model provides a distinct advantage that cellular processes can be analyzed and imaged in vertebrate sensory axons within the intact, living animal. We demonstrate that CMT2b Rab7 mutants cause defects in axon growth, branching and pathfinding in developing sensory neurons. Some of these defects are phenocopied by expression of CA-Rab7, but not by dominant negative Rab7, T22N (DN-Rab7), consistent with a partial gain of function effect of the CMT2b mutants. We also use high speed imaging of vesicle dynamics in developing neurons in vivo and find that CMT2b Rab7 mutants cause reduced vesicle transport speeds, suggesting altered transport may underlie axon development defects. Our data suggest that defects in axon development may be a previously unrecognized component of CMT2b disease.

## Results

### Peripheral sensory neuron outgrowth and branching are reduced in CMT2b Rab7 mutant expressing neurons

**Fig. 1:**
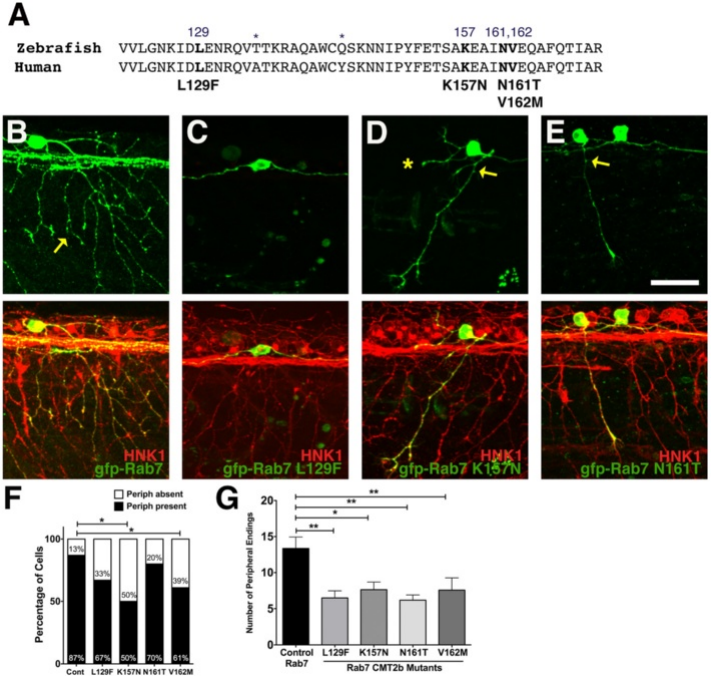
Decreased peripheral axon branching in sensory neurons expressing CMT2b Rab7 mutants. **a** Comparison of zebrafish and human Rab7 protein sequence in the region of CMT2b mutations. Differences in amino acid sequence indicated by asterisks. Amino acid residues associated with CMT2b are bolded. **b**-**e** Confocal images of individual RB neurons expressing wildtype GFP-Rab7 (**b**) or CMT2b GFP-Rab7 mutants (**c**) in embryos with all RB neurons labeled with HNK-1 antibody (*red*). Anterior to the left. **b** wildtype Rab7 expressing cell shows wide arborization of peripheral axons (yellow arrow). **c** Lack of peripheral axon outgrowth in Rab7 L129F expressing cell. **d**, **e** Reduced peripheral branching (yellow arrow) in Rab7 K157N (D) and Rab7 N161T (E) expressing cells. Asterisk = missing central axon. **f** Quantification of number of neurons extending peripheral axons. Cont = wildtype Rab7: *n* = 31 cells in 19 embryos; Rab7 L129F: *n* = 33 cells in 24 embryos, *p* = 0.08; Rab7 K157N: *n* = 10 cells in 10 embryos, *p = 0.03; Rab7 N161T: *n* = 15 cells in 14 embryos, *p* = 0.6; Rab7 V162M: *n* = 23 cells in 18 embryos, *p = 0.05. Fisher’s exact tests. **g** Quantification of peripheral branch endings in CMT2b-associated Rab7 mutations shows a significant decrease in branching. Wildtype Rab7: *n* = 20 cells in 15 embryos; Rab7 L129F: *n* = 21 cells in 18 embryos; Rab7 K157N: *n* = 11 cells in 7 embryos; Rab7 N161T: *n* = 12 cells in 10 embryos; Rab7 V162M: *n* = 16 cells in 14 embryos. *p = 0.03, **p < 0.01; Unpaired two-tailed *t*-test. Scale bar = 40 μm

To investigate the effects of CMT2b mutations in a vertebrate model, we used zebrafish Rohon-Beard (RB) spinal sensory neurons. Zebrafish Rab7 shares 97.6 % amino acid identity with the human Rab7 protein, including the amino acid residues that are affected in the human disease, L129F, K157N, N161T, and V162M [3–5] (Fig. 1a), which makes the zebrafish an excellent vertebrate organism in which to model this disease. Using single-site mutagenesis, we generated these CMT2b disease mutations in the zebrafish *rab7* cDNA and expressed these constructs in RB neurons under control of cis-regulatory elements from the *neurogenin1* gene [20] (-*3.1ngn:GFP-Rab7-cmt2b*). In humans CMT2b is autosomal dominant—the presence of only one copy of the mutated gene causes disease. Thus, we modeled the disease by expressing the CMT2b Rab7 mutants in a background containing wildtype Rab7.

We first expressed the constructs transiently by injecting DNA into 1-cell stage embryos, which results in mosaic labeling of individual sensory neurons, and analyzed the effects on neuronal morphology at 23 hpf, when RB axon arbors are developing. RB neurons have stereotyped morphology; they extend two central axons that ascend and descend ipsilaterally in the spinal cord, and one peripheral axon that extends to the skin where it branches extensively. We first analyzed outgrowth and branching of the peripheral RB axon. Neurons expressing wildtype Rab7 (*n* = 31 cells in 19 embryos) showed normal morphology (Fig. 1b), with most extending a peripheral axon by this stage. Only 13 % of these neurons failed to extend a peripheral axon, which is the same proportion we previously found for wildtype RB neurons expressing only GFP (4 of 31 cells expressing wildtype Rab7, and 3 of 23 cells expressing GFP alone, failed to extend a peripheral axon, *p* = 0.99, Chi-Square test) [21]. This result suggests that overexpression of wildtype Rab7 in the neuron does not induce axon growth defects. In contrast, expression of CMT2b Rab7 mutants caused defects in outgrowth of the peripheral RB axons (Fig. 1c-f). Overall, 20–50 % of RB neurons expressing CMT2b Rab7 mutants failed to extend a peripheral axon. The effects of the four different mutant variants were variable, and only K157N and V162M showed statistically significant effects on peripheral axon outgrowth (Fig. 1f). In addition, the peripheral axons that did form showed decreased branching. We quantified axon branching by counting the number of peripheral axon endings and found a significant reduction in the number of branches in neurons expressing any of the four CMT2b Rab7 mutants (Fig. 1g). These results suggest that CMT2b Rab7 mutants influence both the ability of the peripheral axon to form and its capacity to extend secondary branches.

### Constitutively Active (CA) Rab7 inhibits sensory peripheral axon outgrowth and branching

**Fig 2:**
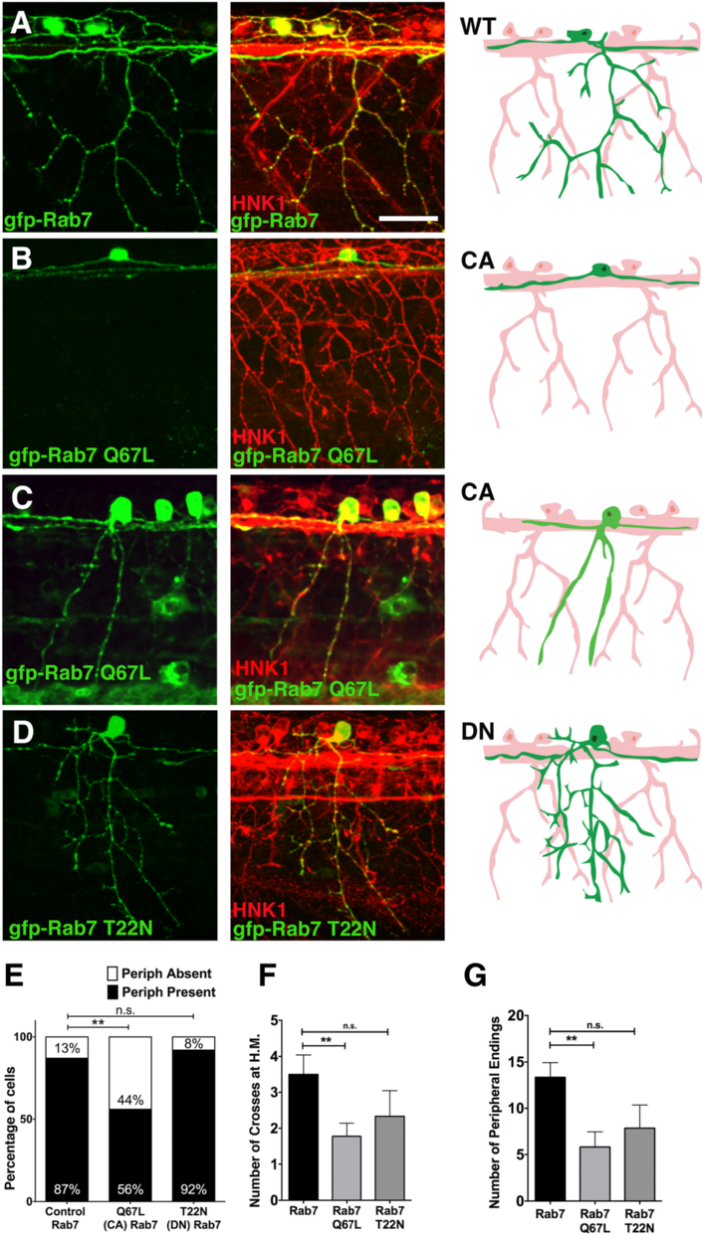
Peripheral axon outgrowth and branching defects in CA-Rab7 expressing neurons. **a**-**c** Confocal projections of embryos with all RBs labeled with HNK-1 antibody (red) and individual RBs labeled with indicated Rab7 forms (*green*). Drawings at right highlight morphology of one neuron in green. **a** Individual RB neuron labeled with GFP-Rab7 showing central axons extending anteriorly and posteriorly from the cell body, and peripheral axon branching in the skin. **b** Lack of peripheral axon outgrowth in GFP-Rab7 Q67L (CA) expressing cell. **c** Reduced peripheral branching in GFP-Rab7 Q67L (CA) expressing cell. **d** Normal central outgrowth and peripheral branching in GFP-Rab7 T22N (DN) mutant expressing RB cell. **e** Expression of CA-Rab7, but not DN-Rab7, mutant construct increases percentage of RB neurons that do not extend a peripheral axon. *n* = 31 cells in Rab7 control, 30 cells in CA-Rab7, and 14 cells in DN-Rab7, **p = 0.006, Chi-Square test. **f** Number of peripheral axons that cross horizontal myoseptum (H.M.) is reduced in CA-Rab7 expressing cells. CA-Rab7: *n* = 9 cells; wildtype Rab7 control: *n* = 12 cells. **p = 0.009, paired *t*-test. **g** Number of peripheral axon endings in individually labeled neurons **p = 0.005, Unpaired two tailed *t*-test. Wildtype Rab7: 31 cells in 19 embryos; Rab7 Q67L: 30 cells in 15 embryos; Rab7 T22N: 14 cells in 9 embryos. All views anterior to the left. Scale bar = 40 μm

To ask whether the effect on RB peripheral axon growth and branching is caused by overactive Rab7 or partial loss of Rab7 function, we analyzed the effects of expressing the previously characterized DN-Rab7 (T22N) or CA-Rab7 (Q67L) [22]. We expressed these constructs under control of the -*3.1ngn1* sensory neuron promoter. Like the CMT2b Rab7 mutants, expression of CA-Rab7 in wildtype embryos caused defects in RB peripheral axon outgrowth and branching. Significantly fewer RB neurons extended a peripheral axon compared to neurons expressing wildtype Rab7 (Fig. 2a-b, e). Further, the peripheral axons that did grow out had fewer branches (Fig. 2c, f-g). In contrast, DN-Rab7 expression had no significant effect on RB axon development (Fig. 2d, e-g). The similarity in phenotype caused by the CMT2b Rab7 mutants and the CA-Rab7 suggest that CMT2b mutant effects on sensory axon development are caused at least in part by Rab7 gain of function.

### CMT2b-associated Rab7 mutants cause central axon guidance defects

**Fig. 3:**
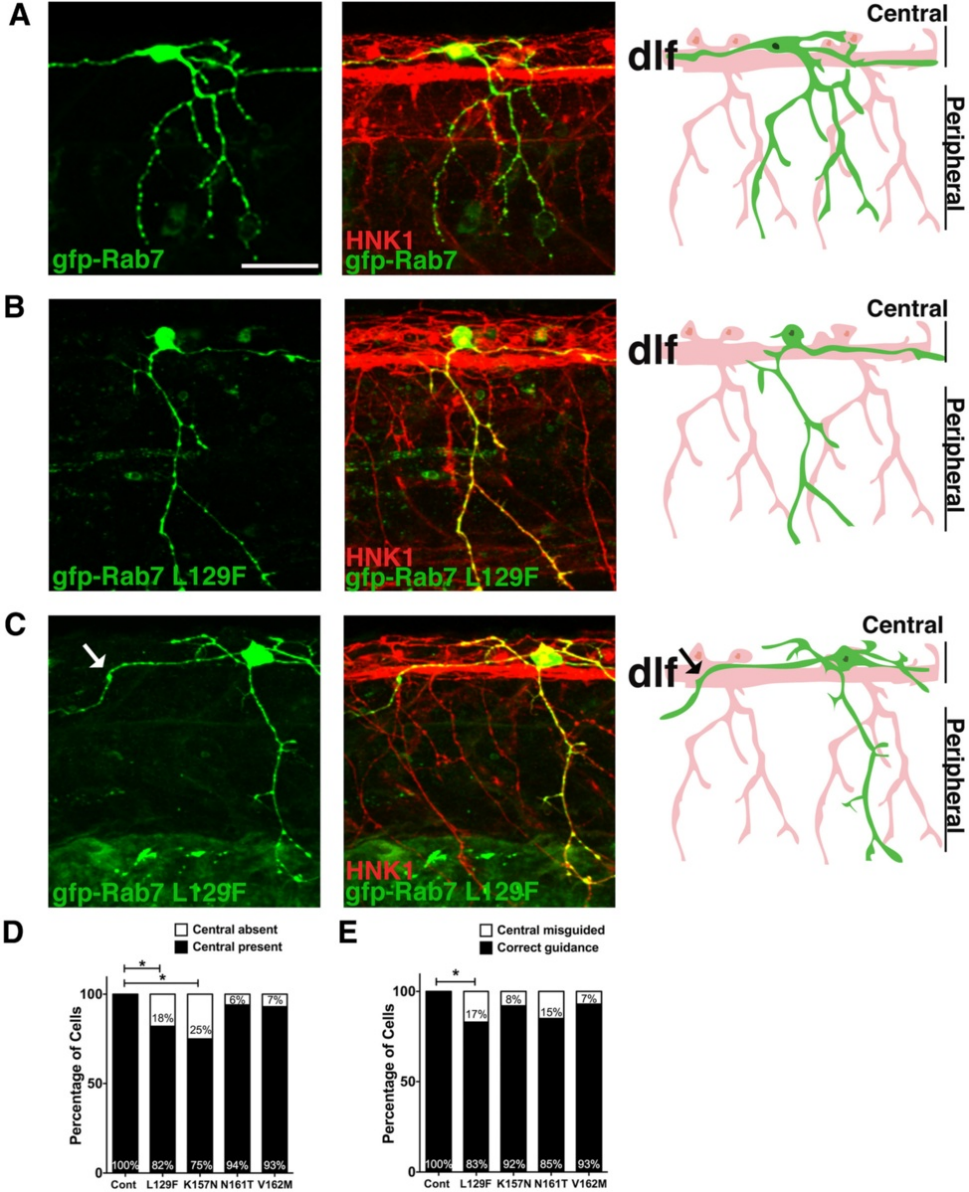
Central axon guidance errors in sensory axons expressing CMT2b Rab7 mutants. **a**-**c** Confocal projections (lateral views, anterior to the left) of embryos labeled with HNK-1 antibody (*red*) and individual RBs expressing GFP-Rab7 forms (*green*). Drawings at right highlight morphology of one neuron in green. **a** Wildtype GFP-Rab7 expressing neuron with two central axons traveling in the dorsal longitudinal fascicle (DLF), and with one peripheral axon branching in the skin. **b**, **c** RB neurons expressing GFP-Rab7 L129F show lack of outgrowth of ascending central axon (**b**) or central axon guidance errors (**c**). Central axon leaving DLF indicated by an arrow in (**c**). **d** Quantification of percentage of neurons lacking a central axon. Cont = wildtype Rab7: *n* = 30 cells in 15 embryos; Rab7 L129F: *n* = 22 cells in 20 embryos, *p = 0.03; Rab7 K157N: *n* = 12 cells in 8 embryos, *p = 0.02; Rab7 N161T: *n* = 16 cells in 11 embryos, *p* = 0.3; Rab7 V162M: *n* = 14 cells in 12 embryos, *p* = 0.3; Fisher’s exact tests. **e** Quantification of percentage of cells with central axon guidance errors. Cont = wildtype Rab7: *n* = 30 cells in 15 embryos; Rab7 L129F: *n* = 24 cells in 19 embryos, *p = 0.03; Rab7 K157N: *n* = 13 cells in 9 embryos, *p* = 0.3; Rab7 N161T: *n* = 13 cells in 11 embryos, *p* = 0.09; Rab7 V162M: *n* = 14 cells in 12 embryos, *p* = 0.3. Scale bar = 40 μm

We analyzed the RB central axon projections and found that expression of CMT2b Rab7 mutants also caused defects in central axon growth and guidance (Fig. 3). All neurons expressing wildtype Rab7 showed normal central axon growth and trajectories (Fig. 3a), as did all neurons expressing GFP alone analyzed previously [21]. In contrast, a significant percentage of neurons expressing L129F and K157N mutants were lacking a central axon (Fig. 3b, d). Further, we found that expression of the L129F Rab7 mutant caused errors in central axon guidance (Fig. 3c, e). In neurons expressing L129F Rab7, central axons left the dorsal longitudinal fascicle and either extended along the ventral spinal cord, or crossed the midline to join the contralateral fascicle. Misguided central axons were seen in sensory neurons expressing any of the four CMT2b Rab7 mutants, but only at significant numbers for L129F. Interestingly, we did not detect central axon defects in neurons expressing either CA-Rab7 or DN-Rab7 (*n* = 30 cells expressing CA-Rab7, *n* = 14 cells expressing DN-Rab7), suggesting this effect is a result of alteration in Rab7 function specifically caused by the disease-related mutation.

### Sensory neurons expressing stable CMT2b Rab7 mutant transgenes exhibit central and peripheral axon defects

**Fig. 4:**
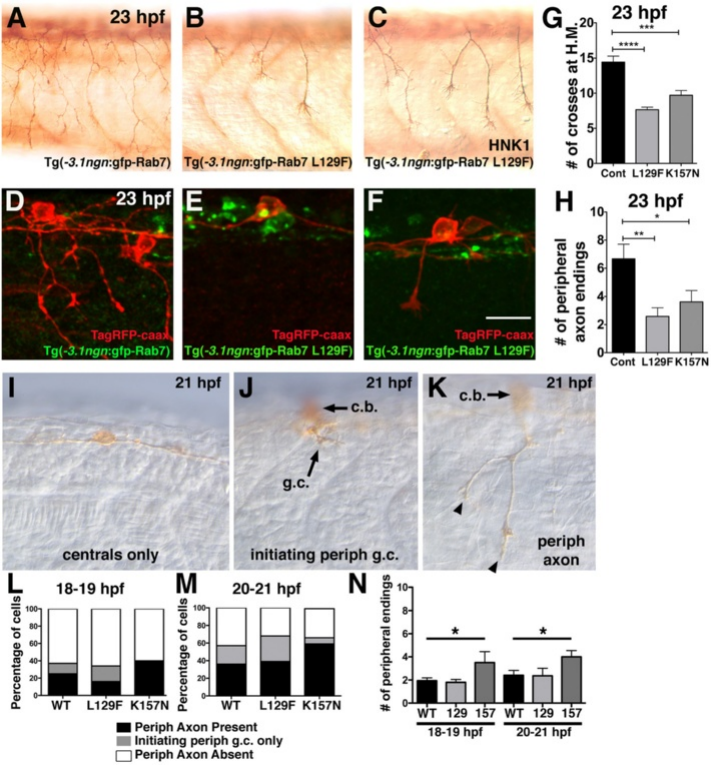
Decreased peripheral axon branching in transgenics expressing CMT2b Rab7 mutants. **a**-**c** Lateral views (anterior to the left) of RB neurons labeled with HNK-1 antibody (*brown*) in 23 hpf transgenic embryos expressing wildtype GFP-Rab7 (**a**) or GFP-Rab7 L129F (**b**-**c**) in all RB neurons, showing decreased branching in Tg(GFP-Rab7L129F) embryos. **d**-**f** Mosaic labeling of single RB cells with TagRFP-caax membrane label (*red*) in 23 hpf transgenic embryos expressing wildtype GFP-Rab7 (**d**) or GFP-Rab7 L129F (**e**, **f**) in all RB neurons. **d** Red-labeled RB in wildtype GFP-Rab7 transgenic embryo with widely branched peripheral axon. **e** Red-labeled RB in GFP-Rab7 L129F transgenic embryo does not extend a peripheral axon. **c** Red-labeled RB in GFP-Rab7 L129F transgenic embryo extends a short peripheral axon that does not branch. Scale bar = 20 μm. **g** Quantification of peripheral branches crossing horizontal myoseptum in 23 hpf Tg(-*3.1*ngn:gfp-Rab7) control (Cont) embryos (*n* = 14 embryos), Tg(-*3.1*ngn:gfp-Rab7 L129F) embryos (L129F, *n* = 63 embryos), and Tg(-*3.1*ngn:gfp-Rab7 K157N) embryos (K157N, *n* = 20 embryos). ****p < 0.0001, ***p = 0.0001. Unpaired, two-tailed *t*-test. **h** Number of peripheral branch tip endings in 23 hpf Tg(-*3.1*ngn:gfp-Rab7) control (*n* = 9 cells in 6 embryos), Tg(-*3.1*ngn:gfp-Rab7 L129F) (*n* = 24 cells in 24 embryos), or Tg(-*3.1*ngn:gfp-Rab7 K157N) (*n* = 21 cells in 15 embryos) embryos is significantly reduced in embryos expressing CMT2b-associated Rab7 mutants. **p = 0.002, *p = 0.04. Unpaired, two-tailed *t*-test. **i**-**k** Lateral views of 21 hpf embryos injected with *ngn:TagRFP-caax* and labeled with anti-TagRFP antibody. **I**, Example of RB with central axons only and no peripheral axon. **j** Example of RB with a peripheral growth cone (g.c.) just initiating (*arrow*). Cell body (c.b.) is out of focus. **k**, Example of RB with short peripheral (with 2 endings, arrowheads) extended out of the spinal cord. Cell body (c.b.) is out of focus. **l**-**m** Quantification of percentage neurons with peripheral axons at 18–19 hpf (**l**) and 20–21 hpf (**m**). There are no significant differences between wildtype and CMT2b Rab7 mutants. At 18–19 hpf: wildtype *n* = 65 neurons, CMT2b L129F *n* = 61 neurons, CMT2b K157N *n* = 10 neurons, *p* = 0.30 Chi-Square test. At 20–21 hpf: wildtype *n* = 75 neurons, CMT2b L129F *n* = 28 neurons, CMT2b K157N *n* = 27 neurons, *p* = 0.15 Chi-Square test. **n**, Analysis of peripheral axon branch endings. At 18–19 hpf and 20–21 hpf, wildtype vs. CMT2b K157N, *p = 0.03 unpaired student’s *t*-test

Transient mosaic expression of plasmid DNA constructs results in variable, and often very high protein expression levels in individual cells. To drive expression at consistent, moderate levels in all RB neurons and thereby more closely model the human disease, we generated stable transgenic lines expressing either GFP-Rab7, GFP-Rab7L129F or GFP-Rab7K157N under control of the -*3.1ngn1* promoter, using the Tol2 transposase system, which typically results in integration of single copies of the transgene [23, 24]. We raised F1 carriers of the transgene and examined F2 offspring for expression of the constructs. We found that most or all RB neurons express the GFP-Rab7s and that GFP fluorescence levels appeared lower than in embryos transiently expressing plasmid DNA. We analyzed the whole RB population by labeling Tg(-*3.1ngn1:GFP-Rab7-CMT2b*) embryos with HNK1 antibody (Fig. 4a-c). We found that similar to cells transiently expressing CMT2b Rab7 mutants, RB neurons in transgenic 23 hpf embryos showed reduced peripheral axon branching compared to those expressing wildtype Rab7. To quantify branching in the population of RB cells, we counted the number of peripheral axon branches that cross the point of the horizontal myoseptum, and found a significant reduction in the embryos expressing the CMT2b Rab7 mutants (Fig. 4g). To analyze individual cell morphology in these embryos, we labeled cells mosaically by injecting -*3.1ngn1:TagRFP-CAAX* DNA into 1-cell stage transgenic embryos that express the CMT2b Rab7 mutants in all RB neurons. We quantified the number of peripheral axon endings in individually labeled RB neurons, and found a significant reduction in branching of neurons in both the L129F and K157N Rab7 mutant transgenics (Fig. 4h).

To ask whether the reduced branching is due to a failure to initiate peripheral axon branches versus decreased stability or maintenance of branches, we analyzed earlier developmental stages. We again mosaically labeled neurons by injecting -*3.1ngn1:TagRFP-CAAX* DNA into transgenic embryos that express the CMT2b Rab7 mutants in all RB neurons. We analyzed embryos between 18–21 hpf, stages when peripheral axons are initiating growth. We categorized individually labeled cells into 3 groups: no peripheral axon (example shown in Fig. 4i), a peripheral growth cone just beginning initiation (e.g. Fig. 4j), or a peripheral axon extended out of the spinal cord (e.g. Fig. 4k). We found no significant difference in peripheral axon initiation between wildtype, CMT2b L129F or CMT2b K157N embryos at either 18–19 hpf or 20–21 hpf (Fig. 4l, m). We further measured the number of peripheral branch endings in the cells that had extended peripheral axons, and found no decrease in initial branch formation in the CMT2b Rab7 mutant expressing embryos, and in fact a statistically significant increase in the CMT2b K157N embryos (Fig. 4n). These data suggest the earliest stages of outgrowth and branching can occur normally in CMT2b Rab7 mutant expressing neurons, perhaps even to an accelerated degree, but these neurons fail to sustain further growth and branching.

**Fig. 5:**
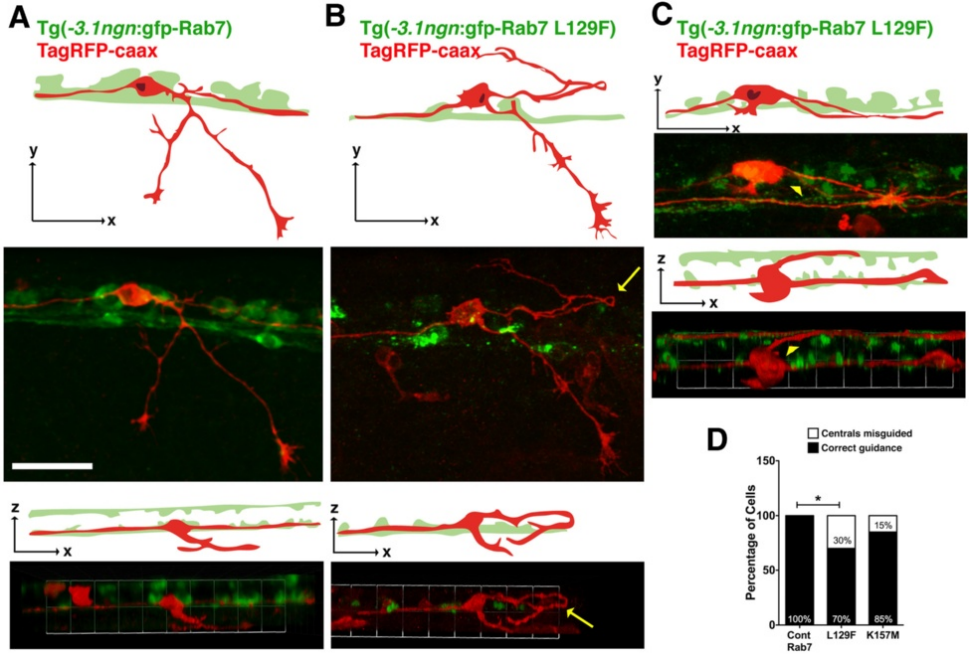
Central axon guidance defects in transgenics expressing CMT2b Rab7 mutants. **a** Lateral (*top two panels*) and 3D rotation dorsal (*bottom two panels*) views of TagRFP-caax labeled RB neuron in Tg(−*3.1ngn*:gfp-Rab7) embryo at 23 hpf showing ascending and descending ipsilateral projections of central axons. Two bilateral rows of RB cells (*green*) are best seen in dorsal views. **b**-**c** Lateral (top two panels) and 3D rotation dorsal (*bottom two panels*) views of TagRFP-caax labeled RB neurons in Tg(−*3.1ngn*:gfp-Rab7 L129F) embryos at 23 hpf. **b** Misguided descending central axon (*yellow arrow*) turns around to travel anteriorly and dorsally from its normal pathway. **c** Misguided central axon (*yellow arrowhead*) crosses dorsal midline to join the contralateral central fascicle. All views anterior to the left. Scale bar = 40 μm. **d** Quantification of number of neurons with central axon guidance errors. Cont Rab7 = Tg(−*3.1ngn*:gfp-Rab7): 10 neurons in 7 embryos; Tg(−*3.1ngn*:gfp-Rab7 L129F): 43 cells in 43 embryos, *p = 0.05; Tg(−*3.1ngn*:gfp-Rab7K157M): 48 cells in 27 embryos, *p* = 0.2; Chi Square test

In addition to peripheral axon branching defects, the L129F and K157M transgenic embryos also exhibited central axon guidance defects (Fig. 5), although only in significant numbers for L129F (Fig. 5d). Guidance defects included axons leaving the central axon fascicle (Fig. 5b), and central axons crossing the midline to join the contralateral central axon fascicle (Fig. 5c). Interestingly, some cells had two central axons that correctly entered the ipsilateral fascicle, with an extra axon that crossed the midline to join the contralateral fascicle of central axons. No guidance defects were found in wildtype Rab7 expressing neurons. Together, our data indicate that in addition to known neurodegenerative effects, the CMT2b Rab7 mutants also cause defects in sensory axon guidance and branch formation during development of the sensory circuitry.

### CMT2b Rab7 transgenics do not show premature RB cell death

**Fig. 6:**
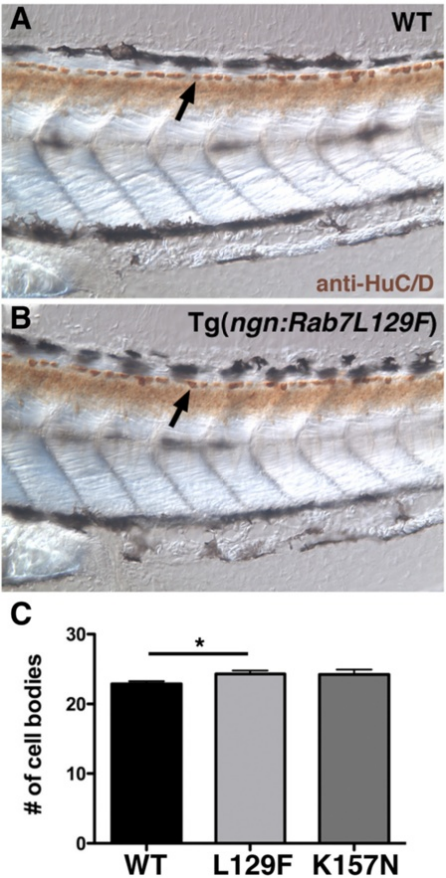
CMT2b Rab7 mutants do not cause increased cell death at 3 dpf. **a**-**b** Lateral views of 3 dpf wildtype (**a**) and CMT2b L129F (**b**) larvae labeled with anti-HuC/D in brown. Arrows indicate RB cell bodies. **c** Quantification of cell body number in five segments beginning at end of the yolk extension. CMT2b L129F is significantly greater than wildtype, *p = 0.02, student’s *t*-test. *N* = 20 embryos for each group

RB neurons normally undergo programmed cell death during larval stages, and their stage of death is regulated in part by neurotrophin signaling [25]. To ask whether the CMT2b Rab7 mutants influence RB cell survival, we analyzed RB cell number at 3 days post fertilization (dpf). We labeled RB cell bodies in 3 dpf embryos with anti-HuC/D, and counted the number of cell bodies in 5 segments beginning at the end of the yolk extension (Fig. 6a, b). We found no reduction in cell number in the CMT2b Rab7 transgenics, and instead found a slight increase in the CMT2b L129F embryos (Fig. 6c). These data suggest that the earlier axon growth defects do not directly inhibit cell survival at later stages.

### CMT2b Rab7 mutants disrupt endosome dynamics in vivo

**Fig. 7:**
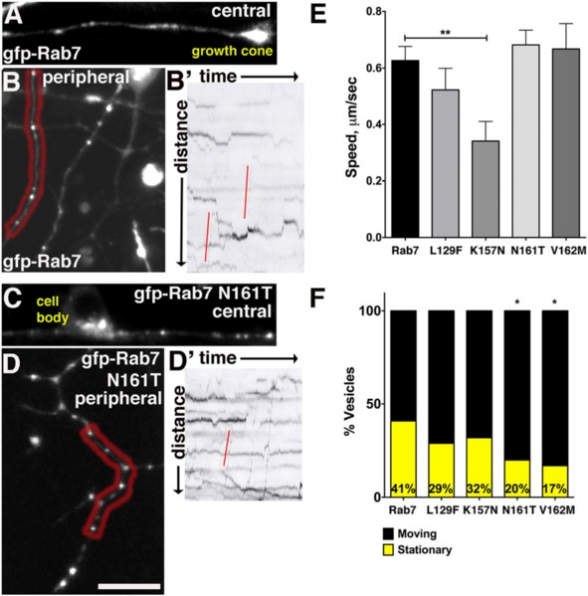
In vivo imaging of CMT2b Rab7 mutant containing vesicles reveals changes in endosome dynamics. **a**-**b** Confocal images of neurons with GFP-Rab7 labeled endosomes in central (**a**) and peripheral (**b**) axons. **b’** Kymograph of peripheral RB axon in red region in (**b**). Red lines indicate rapid retrograde vesicle runs. **c**-**d** Confocal images of neurons with GFP-Rab7 N161T expressing endosomes in central (**c**) and peripheral (**d**) axons. **d’** Kymograph of peripheral axon in red region in (**d**). Red line indicates rapid retrograde vesicle runs. **e** Speeds of vesicles containing Rab7 K157 mutants were significantly reduced in central and peripheral axons. **p = 0.009, Unpaired, two-tailed *t*-test. Wildtype Rab7 control: *n* = 97 vesicles in 8 cells in 6 embryos; Rab7 L129F: *n* = 37 vesicles in 6 cells in 4 embryos; Rab7 K157N: *n* = 24 vesicles in 3 cells in 2 embryos; Rab7 N161T *n* = 73 vesicles in 8 cells in 6 embryos; Rab7 V162M *n* = 33 vesicles in 5 cells in 3 embryos. **f** Decreased percentage of stationary vesicles in central and peripheral axons expressing Rab7 N161T and V162M mutants. *p = 0.01, Fisher’s exact test. Wildtype Rab7 control: *n* = 39 vesicles in 8 cells in 6 embryos; Rab7 L129F: *n* = 86 vesicles in 6 cells in 4 embryos; Rab7 K157N: *n* = 37 vesicles in 3 cells in 2 embryos; Rab7 N161T *n* = 137 vesicles in 8 cells in 6 embryos; Rab7 V162M *n* = 60 vesicles in 5 cells in 3 embryos

Previous studies of CMT2b Rab7 mutant effects on vesicle transport in cultured cells or in *Drosophila* neurons have given diverse results [11, 19]. To explore the effects of CMT2b Rab7 mutants on endosome transport in vertebrate embryos, we performed high speed, high resolution in vivo imaging of vesicle movement using swept field confocal microscopy [26], as we have done previously [21]. We transiently expressed either wildtype GFP-Rab7 or the CMT2b Rab7 mutant constructs by DNA injection at the 1-cell stage, and imaged vesicles in neurons at 24 hpf (Fig. 7). To quantify axonal transport rates of labeled endosomes, we generated kymographs and performed several quantifications of endosome dynamics. Because the average speed of vesicle movement is not representative of dynamic, saltatory movement, we first calculated the speed of the fastest run during a 400 s imaging period. We observed a marked decrease in speed of K157N Rab7-containing vesicles (Fig. 7a-d, e). A previous study found that CMT2b L129F Rab7 mutant vesicles pause less often in *Drosophila* sensory neurons [19]. We also quantified the number of stationary versus moving vesicles, and found fewer stationary CMT2b Rab7 mutant containing vesicles, although this result was only significant for the N161T and V162M Rab7 variants (Fig. 7a-d, f). Finally, to ask if the CMT2b mutants affect vesicle directionality, we analyzed the direction of vesicle movement. We found no significant difference in the directionality of CMT2b mutant Rab7 vesicles (73 % of wildtype Rab7 vesicles moved retrogradely, *n* = 98 vesicles in 4 embryos; 83 % of L129F Rab7 vesicles moved retrogradely, *n* = 35 vesicles in 3 embryos, *p* = 0.4; 66 % of N161T Rab7 vesicles moved retrogradely, *n* = 76 vesicles in 7 embryos, *p* = 0.3; 66 % of V162M Rab7 vesicles moved retrogradely, *n* = 38 vesicles in 4 embryos, *p* = 0.4, Fischer’s exact test).

### Expression of CMT2b Rab7 mutants does not significantly enlarge vesicles in RB sensory neurons

Rab7 is involved in late endosome maturation and formation of lysosomes and autophagosomes [7, 8]. A previous study of cultured cells suggested that CMT2b Rab7 mutants may interfere with Rab7 function in vesicular trafficking and maturation by forming large, vacuolar-like structures, although this effect was seen in PC12 cells, but not in DRG cells [11]. To test the possibility that endosome size is affected by the CMT2b Rab7 mutants in neurons in vivo, we measured the volume of endosomes in central and peripheral axons and found no significant difference in the vesicular volume between wildtype Rab7 and the CMT2b Rab7 mutants (3.8 ± 0.5 μm^3^ in wildtype Rab7, *n* = 85 endosomes; 4.1 ± 0.5 μm^3^ in Rab7 L129F, *p* = 0.1, unpaired, two tailed, *t*-test, *n* = 187 endosomes; 7.0 ± 1.1 μm^3^ in Rab7 K157N, *p* = 0.06, unpaired, two-tailed *t*-test, *n* = 84 endosomes; 4.7 ± 0.9 μm^3^ in Rab7 V162M, *p* = 0.07, unpaired two-tailed *t*-test). These results are consistent with a previous finding that CMT2b Rab7 variants did not interfere with endosomal maturation when expressed in *Drosophila* [15].

## Discussion

In this study we provide the first vertebrate model to investigate the mechanisms by which CMT2b disease associated mutations affect the cell biology of sensory neurons in vivo. We find that expression of CMT2b Rab7 mutants causes developmental defects in vertebrate sensory neurons, including reduced axon outgrowth, reduced axon branching, as well as axon guidance errors. Furthermore, we use live, high-speed, high-resolution imaging to show that CMT2b Rab7 mutants alter endosome transport during axon development in vivo. Overall, these results suggest there may be a developmental component of CMT2b, and that sensory circuits may not form properly in CMT2b disease.

CMT is an unusual form of a neurodegenerative neuropathy, in that it has an early manifestation during the second or third decade of life. Prior studies have suggested that neurodegeneration of long sensory axons are the primary pathological feature of the disease [2, 15, 27, 28], and have suggested that dysregulated neurotrophin trafficking is one of the main factors influencing early neurodegeneration [11, 16]. Our finding that axon development is affected is consistent with previous studies showing that expression of CMT2b Rab7 mutants can inhibit neurite outgrowth in PC12 and Neuro2A cells in culture [11, 18]. In contrast, recent studies of *Drosophila* sensory neurons showed normal size and complexity of dendritic arbors expressing human L129F Rab7 at larval stages [19], and apparently normal development of photoreceptor neurons [15], suggesting neuronal development was not affected in those models. These contrasting results could be due to differences in the dependency on subcellular signaling processes between axons and dendrites, or between different cell types. For example, it is possible that photoreceptors are less dependent on neurotrophin signaling, and axons and dendrites likely exhibit differences in their dependencies on neurotrophin signaling. Our vertebrate sensory axon model may be a better representation of the long sensory axons affected in human disease. Developmental effects of CMT2b are perhaps not surprising, as neurotrophin signaling plays multiple important roles in axon growth and branching, as well as in neuronal survival [29–31]. Moreover, the ability to extend an axon during development could affect the neuron’s ability to receive trophic support. However, we did not see increased RB cell death at 3 dpf, suggesting neurons are still receiving required trophic support. Our results showing that axons fail to maintain branches during development may partly explain the unusually early onset of CMT2b disease, although we do not know if these axon defects directly participate in later axon degeneration.

The effects we see on vesicle dynamics support the idea that disrupted endosomal transport is a component of the disease mechanism. CMT2b Rab7 mutant effects on transport have been reported in other studies, although there are some differences in findings. An in vitro study of DRG neurons showed that vesicles containing CMT2b Rab7 mutants move faster in the anterograde but not retrograde direction [11]. However, in *Drosophila*, the L129F CMT2b Rab7 mutant did not influence average vesicle speed, but did decrease time spent in the stationary phase [19]. We examined all four CMT2b Rab7 mutants, and found varying effects on vesicle dynamics among the individual mutants. We also saw a significant decrease in the number of stationary vesicles containing N161T Rab7 and V162M Rab7 mutants, but not in cells expressing the other CMT2b mutants. In contrast to the Zhang et al. 2013 [11] study, we found that K157N Rab7, but not the other CMT2b mutants, caused a decrease in vesicle speed. A potential explanation for these differing results may be methodological. We quantified the fastest run speed during a specified time period, whereas Zhang et al. quantified the average vesicle speed. However, there also are substantial differences between in vitro and in vivo systems. Developing neurons in vivo are under the influence of the normal repertoire of extracellular signals and guidance cues, including neurotrophin signaling, which undoubtedly affect intracellular processes such as receptor trafficking. Overall, varied results also may be due to the differences in structural changes in the Rab7 proteins induced by individual CMT2b mutations. The L129F substitution does not map to the nucleotide binding pocket, but is predicted to disrupt the positioning of amino acids adjacent to the binding pocket, thus disrupting GTP-GDP cycling. The K157N and N161T substitutions are predicted to cause a regional loss of secondary structure around the binding pocket [12, 13]. Thus, specific CMT2b Rab7 mutants may have variable effects on the ability of vesicles to engage with motors and transport along microtubules. Although differences exist in details of study results, it is apparent that CMT2b Rab7 mutants affect endosomal trafficking, potentially in diverse ways.

The question of whether the CMT2b-associated mutations lead to overactive Rab7 versus loss of Rab7 function has been under debate. It appears likely that a more complex alteration of function is involved. CMT2b Rab7 mutants hydrolyze GTP slower than wildtype Rab7 [12, 13], which has led to the hypothesis that disease effects are caused by overactive Rab7. Indeed several previous studies are also consistent with this hypothesis, as the effects they find can be mimicked with CA-Rab7 but not DN-Rab7 [11, 32–34]. The DN-Rab7 T22N variant interferes with GTP binding, and has dominant negative effects on endosomal trafficking [35]. However, this construct does not have dominant effects in *Drosophila* neurons, suggesting it may not act as a dominant negative under all conditions [15]. CMT2b mutants have reduced binding to both GTP and GDP [12, 13], suggesting these forms may have reduced Rab7 function. In support of this idea, a recent *Drosophila* study showed that CMT2b variants of Rab7 did not have dominant effects and that loss of one *rab7* allele caused defects in photoreceptor synaptic function [15]. These authors conclude that CMT2b disease is in fact caused by reduced Rab7 function. Our results showing decreased peripheral axon branching in both CA-Rab7 and CMT2b Rab7 mutant expressing neurons are consistent with a model in which overactivity of Rab7 contributes to these defects. Interestingly, we found that some aspects of the phenotype caused by CMT2b Rab7 mutant expression in our system, notably, the axon guidance defects, were not phenocopied by either CA-Rab7 or DN-Rab7. This result, together with the diversity of findings from all studies of CMT2b mutants, suggests the CMT2b Rab7 mutant proteins affect multiple cellular processes in distinct ways. The specific effects are likely highly context-dependent, and may vary among cell types and processes (e.g. axon growth or neuronal degeneration) depending on how reliant these processes are on particular signals such as neurotrophin signaling. Continued study of multiple in vivo models will be important for unraveling the mechanisms of this complex disease.

## Conclusions

In this study, we develop the first vertebrate model to investigate the effects of CMT2b-associated alterations in Rab7 protein on long projection sensory neurons, which are the cells most profoundly affected in human disease. We show previously unrecognized effects of CMT2b-associated mutations on early axon development. These results suggest axon developmental defects may be a component of human CMT2b disease.

## Methods

### Animals

Adult zebrafish (*Danio rerio*) were kept in a 14/10 h light/dark cycle. Embryos were maintained at 28.5 °C and staged as hours post-fertilization (hpf) as described [36]. Wild type AB strain or transgenic Tg*(−3.1ngn1:GFP-Rab7)*, Tg*(−3.1ngn1:GFP-Rab7L129F)*, Tg*(−3.1ngn1:GFP-Rab7K157N)* embryos of either sex were used for all experiments. All animal procedures were approved by the Institutional Animal Care and Use Committee at the University of Wisconsin (Animal Welfare Assurance Number A3368-01).

### Immunohistochemistry

Embryos were fixed overnight in 4 % paraformaldehyde and labeled with monoclonal anti-HNK1 antibody (ZN-12, 1:250; Zebrafish International Resource Center, Eugene, OR), anti-HuC/D (1:500, Life Technologies), or with mouse or rabbit anti-GFP antibody (1:1000; Invitrogen, Carlsbad, CA) and rabbit anti-TagRFP antibody (1:500, Evitrogen). Antibody detection was performed with a Vectastain IgG ABC detection kit (Vector Laboratories, Burlingame, CA), or for fluorescent labeling, with AlexaFluor488 and AlexaFluor568-conjugated secondary anti-mouse or anti-rabbit antibodies (4 μg/mL; Invitrogen, Carlsbad, CA).

### Site-directed mutagenesis

The following primers were used to perform PCR-mediated single-site mutagenesis to generate Rab7 L129F, Rab7 K157N, Rab7 N161T, Rab7 V162M, respectively (mutated codon bolded):

- (F) 5’-ccttcaagacactggacag**ttc**gagggatgagtttctgatccagg-3’
- (R) 5’-cctggatcagaaactcatc**cct**cgaactgtccagtgtcttgaagg-3’;
- (F) 5’-gagaccagtgca**aac**gaggccatcaacgtag-3’
- (R) 5’-ctacgttgatggcctc**gtt**tgcactggtctc-3’;
- (F) 5’-gcaaaggaggccatc**acc**gtagagcaggcattcc-3’
- (R) 5’-ggaatgcctgctctac**ggt**gatggcctcctttgc-3’:
- (F) 5’-ggaggccatcaac**atg**gagcaggcattccag-3’
- (R) 5’-ctggaatgcctgctc**cat**gttgatggcctcc-3’

Following PCR-directed mutagenesis, Rab7 constructs were digested with Dpn1 (New England Biolabs) and sequenced.

### DNA constructs and injection

DNA expression constructs were made using Multisite Gateway Cloning System (Invitrogen, Carlsbad, CA) into Tol2 vectors [24]. Rab7, Rab7 T22N (DN) and Rab7 Q67L (CA) constructs in Gateway pDONR vectors [22] were obtained from Brian Link (Medical College of Wisconsin), linked with N-terminal GFP, and cloned behind a cis-regulatory element of the *neurogenin1* gene (-*3.1ngn1*) [20] to drive expression in RB neurons as described previously [37]. To mosaically label RB cells, 11 pg of -*3.1ngn1:TagRFP-CAAX* [37], or 25 pg -*3.1ngn1:GFP-Rab7, -3.1ngn1:GFP-Rab7L129F, -3.1ngn1:GFP-Rab7K157N, -3.1ngn1:GFP-Rab7N161T,* or -*3.1ngn1:GFP-V162M* DNA was injected into one-cell stage embryos.

For transgenesis, AB wildtype embryos at the one-cell stage were co-injected with 25 pg Tol2 transposase mRNA along with 50 pg of either -*3.1ngn1:GFP-Rab7,* -*3.1ngn1:GFP-Rab7L129F* or -*3.1ngn1:GFP-Rab7K157N* DNA. Injected founder embryos were screened for fluorescence and GFP-positive progeny were raised to adulthood.

### Brightfield and fluorescent fixed sample imaging

Brightfield images were captured on a Nikon (Tokyo, Japan) TE300 inverted microscope with a Spot RT camera (Diagnostic Instruments, Sterling Heights, MI). Fluorescent images of fixed embryos were captured with an Olympus (Tokyo, Japan) FV1000 laser-scanning confocal microscope with a 40x (UPlan FLN air, NA 0.75) objective.

### Time lapse imaging

For live confocal imaging, embryos were anesthetized in 0.02 % tricaine and mounted in 1 % low melting agarose in 10 mM HEPES E3 medium as described [38]. Live high speed imaging of endosomal trafficking was performed on a Bruker Opterra swept field confocal microscope (Bruker Nano Surfaces FM, Middleton, WI) equipped with a Nikon CFI Plan Apo VC 60x oil immersion objective (NA 1.40). Embryos were 23 hpf at the beginning of the experiment, and 1–20 1-μm optical sections were captured every 2 s for a total duration of 400 s.

### Quantification and data analysis

Fluorescent images and movies were processed and quantified with Volocity Software (Perkin Elmer, Waltham, MA). For axon growth/branching analysis, the only measurement done in embryos that had all RB neurons labeled with HNK-1 antibody was the counts of axon branches crossing the horizontal myoseptum. Axon crosses over the horizontal myoseptum were counted in somites 8–13 of 23 hpf embryos. All other axon measurements were done on individually labeled neurons (accomplished by mosaic expression of a fluorophore), a technique that allows the entire neuron morphology to be clearly visualized without obstruction from labeled neighboring axons. Neurons were defined as lacking a peripheral axon when the cell body and central axons were visible but no peripheral axon was present. The number of peripheral axon endings were also calculated from individually labeled neurons when the entire arbor including tips of peripheral branches were visible. Axon tips were manually counted from captured images or by examination through the microscope. Axon guidance errors were defined as axons that deviate from the stereotyped pathway and extend into abnormal locations. For cell body number analysis, RB cell bodies labeled with anti-HuC/D were manually counted in a region spanning 5 somite segments, beginning at the end of the yolk extension.

Endosomal trafficking movies were built in Volocity, and corrected for drift in ImageJ [39]. Endosomal speeds were measured in Volocity from kymographs made in ImageJ. Speeds were measured during the fastest run per vesicle in a 400 s imaging period. To determine endosomal direction, we measured the net direction of each individual punctum over 400 s, and categorized vesicle movement as either anterograde (away from the cell body), retrograde (toward the cell body), or no net movement.

All statistical analyses were done using Prism 5.0 (GraphPad Software, Inc.). Errors are reported as standard error of the mean (SEM).

## Abbreviations

CA: constitutively active
CMT2b: Charcot-Marie-Tooth 2b
DN: dominant negative
RB: Rohon-Beard
